# Case report of invasive lobular carcinoma with pineal metastasis

**DOI:** 10.1093/omcr/omag136

**Published:** 2026-07-27

**Authors:** Masayuki Saito, Kanna Ozaki, Hirona Banno, Mirai Ido, Manami Goto, Takahito Ando, Yukako Mouri, Junko Kousaka, Kimihito Fujii, Tsuneo Imai, Shogo Nakano

**Affiliations:** Division of Breast and Endocrine Surgery, Department of Surgery, Aichi Medical University, 1-1, Yazakokarimata, Nagakute-city, Aichi, 480-1195, Japan; Division of Breast and Endocrine Surgery, Department of Surgery, Aichi Medical University, 1-1, Yazakokarimata, Nagakute-city, Aichi, 480-1195, Japan; Division of Breast and Endocrine Surgery, Department of Surgery, Aichi Medical University, 1-1, Yazakokarimata, Nagakute-city, Aichi, 480-1195, Japan; Division of Breast and Endocrine Surgery, Department of Surgery, Aichi Medical University, 1-1, Yazakokarimata, Nagakute-city, Aichi, 480-1195, Japan; Division of Breast and Endocrine Surgery, Department of Surgery, Aichi Medical University, 1-1, Yazakokarimata, Nagakute-city, Aichi, 480-1195, Japan; Division of Breast and Endocrine Surgery, Department of Surgery, Aichi Medical University, 1-1, Yazakokarimata, Nagakute-city, Aichi, 480-1195, Japan; Division of Breast and Endocrine Surgery, Department of Surgery, Aichi Medical University, 1-1, Yazakokarimata, Nagakute-city, Aichi, 480-1195, Japan; Division of Breast and Endocrine Surgery, Department of Surgery, Aichi Medical University, 1-1, Yazakokarimata, Nagakute-city, Aichi, 480-1195, Japan; Division of Breast and Endocrine Surgery, Department of Surgery, Aichi Medical University, 1-1, Yazakokarimata, Nagakute-city, Aichi, 480-1195, Japan; Division of Breast and Endocrine Surgery, Department of Surgery, Aichi Medical University, 1-1, Yazakokarimata, Nagakute-city, Aichi, 480-1195, Japan; Division of Breast and Endocrine Surgery, Department of Surgery, Aichi Medical University, 1-1, Yazakokarimata, Nagakute-city, Aichi, 480-1195, Japan

**Keywords:** breast cancer, invasive lobular carcinoma, pineal metastasis, radiation therapy, comprehensive genomic profiling

## Abstract

A 59-year-old woman was diagnosed with luminal-type invasive lobular carcinoma (ILC) of the breast, cT3N3bM0, cStage IIIC. After neoadjuvant chemotherapy, she underwent mastectomy and axillary lymph node dissection, and pathological examination revealed ypT3N0M0, ypStage IIB. She subsequently received postmastectomy radiation therapy and endocrine therapy. Nine months after surgery, she developed multiple bone metastases, followed one month later by headache and diplopia. Brain magnetic resonance imaging revealed lesions in the occipital lobe, cerebellum, and pineal gland. Endoscopic biopsy confirmed pineal metastasis of ILC with subtype conversion to triple-negative breast cancer. Whole-brain radiation therapy with local boost irradiation was administered, followed by systemic chemotherapy. Comprehensive genomic profiling revealed high tumor mutational burden. This case highlights the clinical value of biopsy for atypical brain metastases in guiding therapeutic decision-making.

## Introduction

Distant metastases of breast cancer commonly occur in the bones, lungs, liver, and brain. Among malignant tumors, brain metastases from breast cancer are the second most common after lung cancer [1]. Among the histologic subtypes of breast cancer, invasive lobular carcinoma (ILC) has a greater tendency to metastasize to unusual sites than invasive ductal carcinoma (IDC) [2].

Pineal gland tumors are uncommon, with an incidence of less than 1% of all brain tumors diagnosed in adults. Pineal gland tumors are classified into three main categories: germ cell tumors, pineal parenchymal tumors, and pineal metastases. The diagnosis is based on clinical symptoms, evaluation of biomarkers in the blood and cerebrospinal fluid, imaging studies, and tissue biopsy. The treatment strategies employed vary depending on the specific type of primary pineal tumor, as well as on whether the tumor is primary or metastatic. Therefore, establishing a definitive diagnosis is essential, and a tissue biopsy is required [3].

Here, we present a case of pineal gland metastasis from ILC.

## Case report

A 59-year-old woman presented with a palpable lump in her left breast in January of year X − 1 and visited a local physician one month later. Her medical and family histories were unremarkable for malignancies. Systemic evaluation led to the diagnosis of ILC, cT3N3bM0, cStage IIIC, estrogen receptor (ER) 50%, progesterone receptor (PgR) 50%, human epidermal growth factor receptor 2 (HER2) 1+, and Ki-67 10%. She received neoadjuvant chemotherapy with four courses of epirubicin (90 mg/m^2^) and cyclophosphamide (600 mg/m^2^), followed by four courses of docetaxel (75 mg/m^2^). In August, she underwent a left mastectomy and axillary lymph node dissection. Pathological findings revealed ILC, ypT3N0M0, ypStage IIB, ER 40%, PgR 0%, HER2 0, Ki-67 5%, and therapeutic grade 2a. Following surgery, postmastectomy radiation therapy (50 Gy, 25 Fr) and adjuvant endocrine therapy with anastrozole (1 mg/day) and abemaciclib (300 mg/day) were initiated. However, abemaciclib was discontinued after three months because of adverse events, including diarrhea and renal dysfunction. Nine months after surgery, she presented with right thigh pain and was diagnosed with multiple bone metastases after evaluation for a pathological femoral fracture. Total joint replacement surgery was performed for the fracture and denosumab therapy was initiated. One month after the diagnosis of bone metastases, she developed headaches and diplopia. Gadolinium-enhanced MRI revealed tumorous lesions in the occipital lobe, cerebellum, and pineal gland, measuring up to 11, 5, and 20 mm, respectively. Ventricular enlargement and peritumoral edema were observed around the pineal lesion (Fig. 1). The patient presented with headache and diplopia, which were attributed to hydrocephalus and midbrain compression affecting the third cranial nerve, as demonstrated by MRI. Following multidisciplinary discussion with radiologists and neurosurgeons, multiple brain metastases were suspected; however, metastasis to the pineal gland was considered extremely rare. An endoscopic pineal tumor biopsy was planned to differentiate metastatic disease from a primary brain tumor, and an endoscopic third ventriculostomy (ETV) was scheduled because of ventricular enlargement. The biopsy results showed ILC, ER 0%, PgR 0%, HER2 1+, and Ki-67 10% (Fig. 2). Compared with the primary tumor (ER 40%–50%, PgR 0–50%, HER2-low), the pineal lesion demonstrated complete loss of hormone receptor expression (ER 0%, PgR 0%) while maintaining HER2-low status. PD-L1 (SP142, 22C3) expression was negative. Germline *BRCA* testing was performed but no pathogenic variants were found (Myriad, BRACAnalysis$\mathrm{R}$). Two months after the diagnosis of bone metastasis, whole-brain radiation therapy (WBRT) with hippocampal dose reduction and local boost irradiation (30 Gy, 10 Fr) was administered. In July, treatment for the recurrence was started using eribulin (1.4 mg/m^2^). The diplopia symptoms improved, and gadolinium-enhanced MRI performed three months later demonstrated shrinkage of the metastatic lesions, indicating a partial response (Fig. 3). One month after initiating eribulin, treatment interruption was required because of fever and pneumonia. Subsequent computed tomography revealed progression with new liver metastases, and treatment was changed to bevacizumab (Bev, 10 mg/m^2^) plus paclitaxel (PAC, 80 mg/m^2^). We performed comprehensive genomic profiling (CGP) using the OncoGuide™ NCC OncoPanel System$\mathrm{R}$, which demonstrated a high tumor mutational burden (TMB, 13.2 mutations/Mb) and somatic mutations in *TP53*, *PIK3CA*, *NF1*, *FGF4*, and *SMAD4*. Pembrolizumab was identified as a recommended treatment option. Soon after initiating Bev and PAC, and following consultation with the patient, we planned to administer pembrolizumab after disease progression. However, the patient died seven months after the diagnosis of bone metastasis due to the rapid worsening of liver metastases.

**Figure 1:**
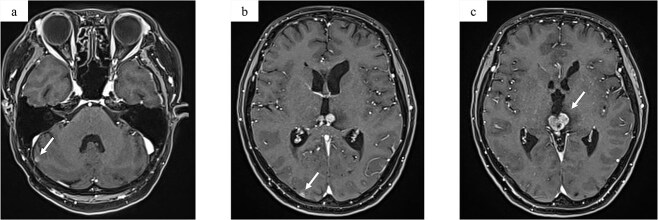
Gadolinium-enhanced head magnetic resonance imaging (MRI) findings. The tumor area is indicated by the arrowhead. (a) cerebellum, (b) occipital lobe, (c) pineal gland. Peritumoral edema and ventricular enlargement were observed around the pineal tumor. In addition, we have changed the color of the arrowhead from yellow to white.

**Table 1:** Comparison of previously reported cases and the present case.

	Age	Histological type	Subtype	Period from diagnosis to pineal metastasis	Symptoms	Metastasis to other organs	Surgical treatment for pineal metastasis	Radiation therapy	Systemic therapy	Prognosis
S. Kanoun Belajouza et al. [11]	76	Invasive ductal carcinoma	Luminal type	Approximately 3 years	Headache, ptosis of the right eyelid	Cavernous sinus, multiple bones, bilateral adrenal glands	None	Whole-brain radiation	Hormone therapy	Survival
Our case	59	Invasive lobular carcinoma	Primary tumor: luminal type Pineal tumor: triple negative type	Approximately 1.5 years	Headache, double vision	Multiple bones, occipital lobe, cerebellum	Pineal region biopsy and endoscopic third ventriculostomy	Whole-brain radiation	Chemotherapy	Died approximately six months after the pineal metastasis diagnosis

**Figure 2:**
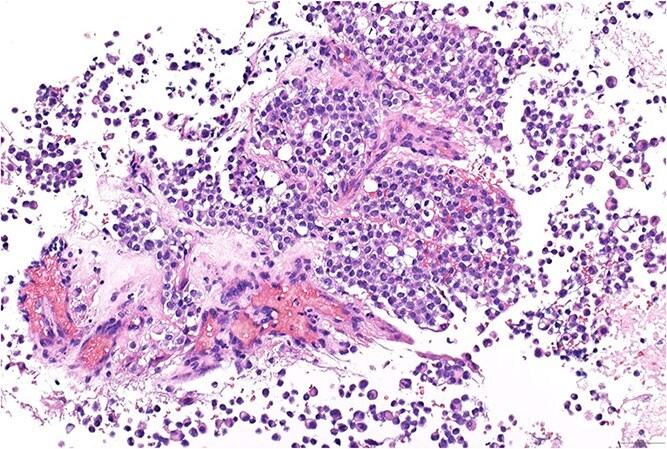
Histopathology image of the pineal tumor biopsy. Hematoxylin–eosin staining, ×200 magnification.

**Figure 3:**
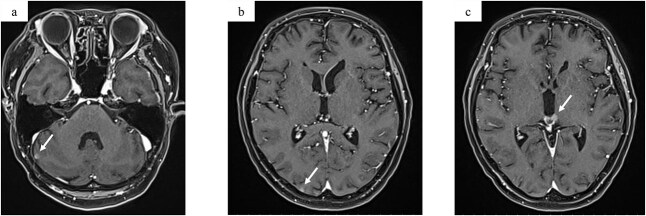
Gadolinium-enhanced head magnetic resonance imaging (MRI) findings three months after whole-brain irradiation with hippocampal dose reduction plus local boost irradiation (30 Gy, 10 fractions). All metastatic lesions decreased in size. The tumor site is indicated by an arrow. (a) cerebellum, (b) occipital lobe, (c) pineal gland. In addition, we have changed the color of the arrow from yellow to white.

## Discussion

The principal clinical implication of this case is that biopsy of an atypical pineal lesion provided clinically actionable information beyond diagnostic confirmation alone. Histological confirmation of metastasis, identification of subtype conversion from luminal-type breast cancer to triple-negative breast cancer (TNBC), and subsequent CGP collectively influenced therapeutic decision-making.

ILC accounts for approximately 10%–15% of breast cancers and is known to metastasize to uncommon sites more frequently than IDC [4]. Pineal metastasis is particularly rare, and radiological differentiation from primary pineal tumors can be challenging [3, 5–7]. In this case, the pineal lesion was accompanied by hydrocephalus and multiple brain metastases; however, because metastasis to the pineal gland is uncommon, endoscopic biopsy was performed not only to obtain a definitive diagnosis but also to relieve obstructive hydrocephalus through an endoscopic third ventriculostomy. This approach yielded clinically meaningful information beyond diagnostic confirmation alone.

Re-biopsy of metastatic lesions may be particularly important when the clinical course, metastatic pattern, or treatment response appears discordant with the biological characteristics of the primary tumor. In this case, reassessment of the pineal lesion demonstrated receptor discordance with complete loss of hormone receptor expression, resulting in subtype conversion from luminal-type disease to TNBC. Receptor discordance between primary and metastatic breast cancer is increasingly recognized and may substantially affect treatment selection [8–10]. Therefore, tissue reassessment should be considered when treatment modification is anticipated or when atypical disease progression is observed.

Only a limited number of breast cancer cases with pineal metastasis have been reported [11–13]. In previously reported cases, molecular reassessment of metastatic lesions was limited. In contrast, this case underwent biopsy confirmation, subtype reassessment, and CGP using metastatic tissue. Compared with previous reports, this case highlights the potential clinical value of obtaining tissue from atypical brain lesions when the results may influence systemic therapeutic strategies.

CGP identified TMB-high status and mutations in *TP53*, *PIK3CA*, *NF1*, *FGF4*, and *SMAD4* genes, suggesting pembrolizumab as a potential therapeutic option despite negative PD-L1 expression [14, 15]. However, actionable findings were obtained only after disease progression had already occurred. Limited tissue volume from the pineal biopsy may have contributed to prolonged turnaround time. Therefore, this case highlights an important practical consideration: CGP may be useful when performed sufficiently early to allow implementation of genomically informed treatment strategies, particularly in patients with rapidly progressive disease.

The prognosis of pineal metastasis is generally poor, with a survival time of one to three months [7, 12, 13]. In this case, local symptoms, including headache and diplopia attributed to hydrocephalus and midbrain compression of the third cranial nerve, improved following biopsy and WBRT with local boost irradiation, leading to a radiological partial response of the brain lesions. Although definitive conclusions cannot be drawn from a single case, early local intervention followed by reassessment of tumor biology may have contributed to temporary disease control and subsequent systemic treatment selection.

Overall, this case suggests that tissue reassessment of atypical brain metastases may support therapeutic decision-making by identifying clinically relevant biological changes. Histological reassessment and CGP may support treatment selection in selected patients with metastatic breast cancer, although timing, tissue availability, and disease progression remain important practical considerations.

## Conclusions

In conclusion, we report a case of pineal gland metastasis from ILC in which endoscopic biopsy provided important information for treatment selection beyond diagnostic confirmation alone. Histological reassessment of the atypical brain lesion revealed subtype conversion from luminal-type disease to TNBC and enabled CGP, both of which influenced therapeutic decision-making. This case suggests that tissue confirmation and re-biopsy should be considered in selected patients with atypical metastatic patterns or discordance between clinical behavior and previously known tumor biology.
